# Estimating anatomically plausible segment orientations using a kinect one sensor

**DOI:** 10.1080/07853890.2021.1896569

**Published:** 2021-09-28

**Authors:** Nuno Vaz Matias, Ivo Roupa, Sérgio Gonçalves, Miguel Tavares da Silva, Daniel Simões Lopes

**Affiliations:** a INESC-ID, Instituto Superior Técnico, Universidade de Lisboa, Lisboa, Portugal; b IDMEC, Instituto Superior Técnico, Universidade de Lisboa, Portugal

## Abstract

**Introduction:**

Marker-based motion tracking systems are the golden standard for human motion analysis, however such systems are expensive, non-portable and require long time subject preparation. The Kinect One sensor, being inexpensive, portable and markerless, appears as a reliable and valid alternative to the marker-based systems in several situations [1–3]. This sensor acquires depth image data and colour camera data that are processed by a tracking algorithm to estimate the three-dimensional position of twenty-five anatomical joints in real-time [4]. Nevertheless, the internal orientations of each anatomical segment are poorly estimated. The main objective of this work is to study the effectiveness of vector orthogonalization methods to estimate the relative internal orientations of the anatomical body segments using the skeletal data acquired by a Kinect One sensor.

**Materials and methods:**

Twenty-eight young healthy adults (25 ± 9 yrs old, 170 ± 9 cm height, 61 ± 9 kg weight, 13 women) performed 5 repetitions of ten different elementary movements: shoulder flexion/hyperextension, shoulder abduction/adduction, shoulder transversal abduction/adduction, shoulder medial/lateral rotation, elbow flexion, forearm pronation/supination, hip flexion/hyperextension, hip abduction/adduction, knee flexion and hip medial/lateral rotation. On each repetition, the subject initiated the movement in an adapted pose of the anatomical reference position and once finished returned to the initial position. Data was collected, simultaneously, using a marker-based system (Qualysis − 100 Hz) and a markerless system (Kinect One − 30 Hz). All participants signed consent forms.The biomechanical model used was composed by eleven anatomical segments: the head, the chest, the abdomen and both arms, forearms, thighs and legs. Six different vector orthogonalization methods (Householder, Eberly, Square Plate, Spherical and Projection Matrix) were used to estimate the relative orientations of the anatomical body segments from Kinect One sensor model [5]. Pearson’s correlation coefficient was used to compare the anatomical body segments orientations of all model segments obtained with both systems.

**Results:**

The results obtained show that the six techniques implemented present a moderate to high correlation (0.58 − 0.93) between segments longitudinal axis of rotation while for the remaining axes (anterior-posterior and medial-lateral) they show a moderate to negligible correlation (–0.37 to 0.46). Additionally, the performance of each technique varies according the selected movement. For example, the Householder technique presents different correlation values when applied to the following movements, hip flexion (0.84), hip abduction (–0.05), knee flexion (0.78), shoulder flexion (0.36), elbow flexion (0.80) present relevant differences.

**Discussion and conclusions:**

Although vector orthogonalization techniques are capable to estimate plausible orientations, the results given the same movement shows significant differences, suggesting that not all vector orthogonalization techniques are appropriate for all movements. Therefore, it is necessary to careful select the best technique for each movement in order to obtain valid results. Finally, it is possible to conclude that Kinect One shows good results for some kinematic variables, nevertheless, it needs to improve the precision on the estimation of the joints’ position and all body segments’ orientation in order to obtain results similar to marker-based systems.
